# From Fragmentation to Intensification: Land Reform in China’s “New Era”

**DOI:** 10.3390/ijerph191811223

**Published:** 2022-09-07

**Authors:** Qiang Wang, Liying Yu, Yueling Yang

**Affiliations:** School of Business, Shandong Management University, Jinan 250357, China

**Keywords:** Land Reform, willingness to transfer land (WTT), willingness to the duration of land transfer (WTD)

## Abstract

Combining the current national conditions of China and the status quo of rural land, realizing the transformation of land from fragmentation to intensification is the only way for China to move towards agricultural modernization. We selected Feicheng City, Shandong Province, as the research area, conducted regression analysis on the data by means of questionnaires and key interviews, and identified the influencing factors that can affect and change farmers’ willingness to transfer (WTT) their land and willingness to the duration (WTD) of land transfer. The study found that 82.54% of farmers are willing to transfer land, and the WTD is 9.34 years. Among them, five factors, including job stability, purchased houses in urban area, cultivated land roads, degree of policy understanding, and emotion for the land, can significantly affect the farmers’ WTT. Six factors, namely, age, job stability, number of family members, purchased houses in urban area, non-agricultural income, emotion for the land, can significantly affect the farmers’ WTD. Based on this, we propose the “MPEU theory” of farmers’ land transfer. That is, by allowing farmers to change their minds, understand policies, increase the non-agricultural employment rate, and improve the level of urbanization, the farmers’ WTT/WTD can be improved, and the level of land intensification can be improved. Finally, agricultural modernization, peasant citizenization, and rural urbanization will be realized.

## 1. Introduction

In 1978, the household contract responsibility system, which was implemented by the Chinese government, played a positive role in China’s agricultural development. The land system extracts the contract rights of collectively owned farmland in rural areas to farmers, which greatly releases the production efficiency of farmers [1]. China’s grain production has risen from 609.54 billion catties in 1978 to 1365.7 billion catties in 2021 [2]. This system has promoted the rapid growth of China’s rural economy and is recognized as a historic leap in the history of China’s agricultural development.

After years of development and changes in national conditions, the disadvantages of the household contract responsibility system have gradually emerged. First, the development of urbanization in China has brought a large number of young rural laborers into cities [3]. As a result, most of the land for small-scale farming by households under the household contract responsibility system has been abandoned [4]. Second, the system came into being based on the relatively low level of agricultural productivity and mechanization at that time. This small-scale and relatively scattered land pattern is neither conducive to the use of large-scale agricultural machinery, nor does it have the value of allowing farmers to invest in agricultural machinery [5].

Agricultural modernization through land intensification is an effective path for rural development. First of all, China has a large number of top talents with professional agricultural knowledge and technology. They master advanced modern agricultural management techniques and concepts. Only by realizing land intensification can the land be concentrated in the hands of professionals for professional operation [6]. Secondly, only by realizing the intensification of land can capital really enter, large-scale machinery and equipment can be used, and the level of agricultural modernization can be greatly improved. This then improves the efficiency of the land scale to achieve multiple benefits for land owners, contractors, and operators [7].

Affected by various factors, farmers are reluctant to transfer their land, which has become a major obstacle in the process of land intensification. Farmers’ willingness to transfer land with land contract rights has an important impact on the level of land intensification [8]. On the one hand, capital has the willingness to intensify land investment, hoping to obtain benefits through large-scale, mechanized, and high-tech models [9]. On the other hand, it is necessary to ensure that farmers’ land income remains unchanged or even higher [10]. In 2014, the Chinese government proposed to adhere to the collective ownership of rural land and complete the “separation of ownership, contract rights and management rights” [11]. This provides policy support for rural land transfer [12].

In countries with private ownership of land, it mainly involves research on changes in the rural land market and system, as well as research on land transactions. The government’s macro-control function on the land market makes the operation of the land market inefficient [13]. The information system of land transfer should be improved, and the land transaction system should be improved by clarifying the land price [14]. Institutional innovation should be used to optimally allocate the vast amount of rural land in such a way that it can improve the efficient use of the land [15]. On the one hand, the proper transfer of land ownership and use rights can improve the resource allocation rate of land, and on the other hand, it can promote in-depth investment in land resources and reduce farmers’ risk aversion behavior to a certain extent [16].

China is dominated by the public and collective ownership of land, and since China’s reform and opening up in 1978, land transfer has changed from prohibition to encouragement, and the policy orientation has gradually become clearer. Domestic scholars in China have carried out a lot of research on how to revitalize rural collective land to better adapt to China’s economic development, and have achieved fruitful results. In the current land transfer, there are problems such as small transfer scale, irregular transfer, and short transfer time [17]. There are also problems such as imperfect market development, imperfect management and management systems, and backward farmers’ concepts [18]. The WTT is affected by factors such as the age of the head of the household, the education level of the head of the household, the size of the household, the area of land managed per household, the proportion of income from planting, and the farmers’ understanding of the policies on agriculture [19]. Age, education level, family non-agricultural income, and pension security also have a significant impact on farmers’ WTT [20].

The joint production contract responsibility system with households as the production unit can no longer meet the needs of current rural development. It has become a trend to transform the traditional small-scale farmer operation mode to a large-scale operation mode, and the time for land transfer and moderate-scale operation is becoming more and more mature. In 2017, the Chinese government proposed the concept of “Socialism with Chinese Characteristics for a New Era”. China in the “new era” further emphasizes the “separation of three rights” of rural land ownership, contract rights, and management rights. Special emphasis is placed on promoting the orderly transfer of management rights while retaining the contracting rights of farmers, realizing the optimal allocation of land resources, and promoting various forms of moderate-scale operation. Therefore, in the context of China’s “new era”, it is particularly urgent to understand farmers’ willingness to transfer land, identify the influencing factors to improve farmers’ willingness to transfer land and the duration of transfer, and maximize the benefits of land.

## 2. Methodology

### 2.1. Research Regions

**Figure 1:**
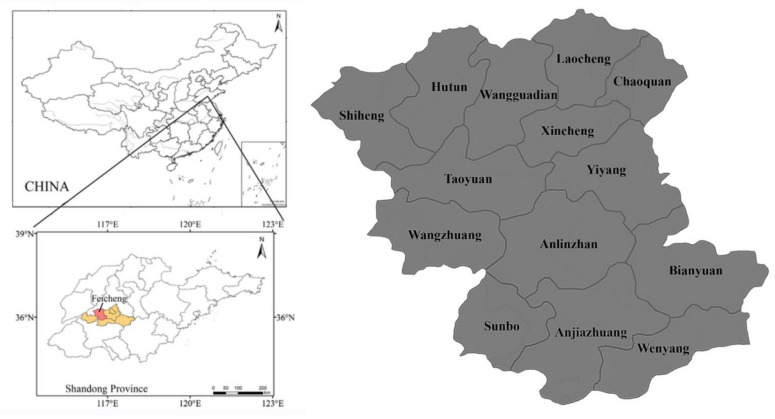
Location map of the study area.

This paper takes Feicheng City, Shandong Province, as the research area (Figure 1), mainly for the following reasons. First of all, Feicheng City is the most representative of the geographical and human characteristics. The city is located in the North China Plain and belongs to the central region of Shandong Province. Its economic development level, living conditions, and cultural customs are the compromise areas of Shandong Province. Secondly, Feicheng City has the feasibility of investigation. At present, its circulating land area is 386,000 Chinese mu, accounting for 48.3% of the total contracted area, with more than 320,000 Chinese Mu of arable land under trusteeship, and a large-scale operation rate of 91% [21]. Therefore, it is highly feasible to choose Feicheng City as the research area. Third, Feicheng City has abundant sample types and sample sizes, so sampling data science. Mountains, hills, and plains each account for about one-third of Feicheng City, with a total cultivated land of 739,300 Chinese Mu [22]. There are many types of agricultural land such as cultivated land, gardens, forest land, and unused land. The resident population is 894,115, and the population living in rural areas is 293,249, accounting for 32.80% [23]. Therefore, this research area is representative, feasible, and scientific.

### 2.2. Questionnaire Design

After referring to a large number of relevant studies, we formulated the survey plan and determined the survey indicators based on the actual situation of Feicheng City, and then designed the survey questions around the indicators, forming the first draft of the questionnaire. Before finalizing the draft, we communicated with experts on rural land policy in universities in Shandong Province, and the staff of Feicheng Agriculture Committee and Agriculture Bureau to solicit their opinions and suggestions. Afterwards, a pre-investigation was conducted in rural areas of Feicheng City. Based on the feedback from the pre-research, we optimized the questionnaire. Because the study mainly deals with farmers, we attempted to make the problem as easy to understand as possible. WTT/WTD, the two most important questions, were added to the last part of the survey (Table 1). In the specific research process, the researcher should ensure that the respondents can complete the research independently without external interference. The time taken to complete each questionnaire was controlled to within 20 min.

The first part is to investigate the farmers’ willingness to transfer land (WTT) and willingness to the duration of land transfer (WTD). The first question is whether the farmers have the willingness to transfer the land, and the second is whether they intend to transfer the land, how long are they willing to transfer the land for. If the farmer is unwilling to transfer the land out, what are the obstacles? The second and third parts are the survey of personal characteristics and family characteristics, respectively. These sections attempt to determine the relationship between the surveyor’s personal situation and family characteristics and the WTT. The fourth part investigates the characteristics of farmers’ land, including the average unit land area, annual land income, and other factors. It is hoped that this information will help to explore the relationship between land characteristics and farmers’ WTT from the perspective of land fragmentation. The fifth part is a survey of farmers’ cognition. This part investigates farmers’ understanding of land transfer policies and their emotional dependence on their land. The purpose of this part of the questionnaire is to grasp the current attitude and cognition level of farmers towards land transfer, so as to propose breakthrough solutions and suggestions for the current land transfer work in China.

### 2.3. Minimum Sample Size

The formula for calculating the sample size is as follows [35,36]:

(1) $$n=\frac{N}{1+N{ɛ}^{2}}$$

(2) $$ɛ=\frac{\rho e}{t}$$

where

*n* = minimum returned sample size,

N = population size,

ɛ = adjust margin of error,

e = the degree of accuracy expressed as a proportion,

ρ = the number of standard deviations that would include all possible values in the range,

t = t-value for the selected alpha level or confidence level at 90%.

We set the confidence interval to 90% and the degree of accuracy to 3%. The number of standard deviations that would include all possible values in the range is 4. By looking at the table, we observe t = 1.645 under the 90% confidence interval. The minimum sample size can be calculated as 128. This is basically consistent with the study of Adam et al. [37].

### 2.4. Data Collection

First of all, through the literature research method to check the “Feicheng County Chronicle”, we logged on the government websites at all levels to check the documents and statistical data to obtain a preliminary understanding of the cultivated land and land transfer in Feicheng City. The sample was then selected. The survey objects of this study involve more than 60 villages and hundreds of respondents in 15 townships and sub-districts under the jurisdiction of Feicheng City. The survey samples covered all townships under the jurisdiction of Feicheng City, and the proportion of the survey samples of each township to the total number of surveys was determined based on the proportion of the total population of each township to the total population of Feicheng City, and the overall distribution was uniform. All respondents were selected by random sampling. The survey time covered January to February 2022. More than 60 villages in 15 townships and streets were investigated, 400 questionnaires were distributed, 400 questionnaires were returned, and 355 valid questionnaires were obtained. During the investigation, the author also conducted key interviews with some township cadres and village cadres to understand the current basic situation of farmland transfer, and listened to their opinions and suggestions on land transfer.

### 2.5. Hypothesis

**Hypothesis** **1** **(H1).** *Farmers’ land transfer behavior is affected by some personal characteristics.*

**Hypothesis** **2** **(H2).** *Farmers’ land transfer behavior is affected by some family characteristics.*

**Hypothesis** **3** **(H3).** *Farmers’ land transfer behavior is affected by some land characteristics.*

**Hypothesis** **4** **(H4).** *Farmers’ land transfer behavior is affected by some cognitive characteristics.*

## 3. Data Analysis

### 3.1. Variable Definitions

The independent variables include four dimensions of farmers’ personal characteristics, family characteristics, land characteristics, and cognitive characteristics. The definitions and assignments of the selected variables and indicators are shown in Table 2.

### 3.2. Descriptive Analysis

**Figure 2:**
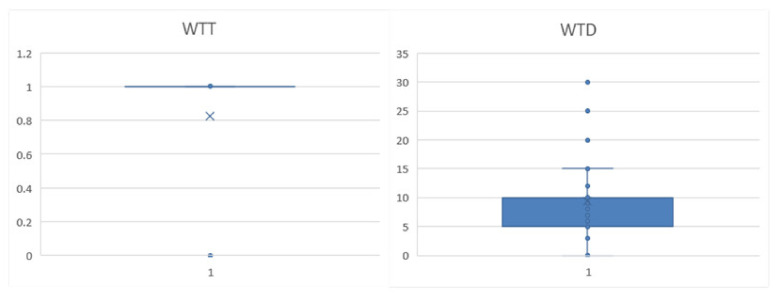
Boxplots of dependent variables descriptive analysis.

**Figure 3:**
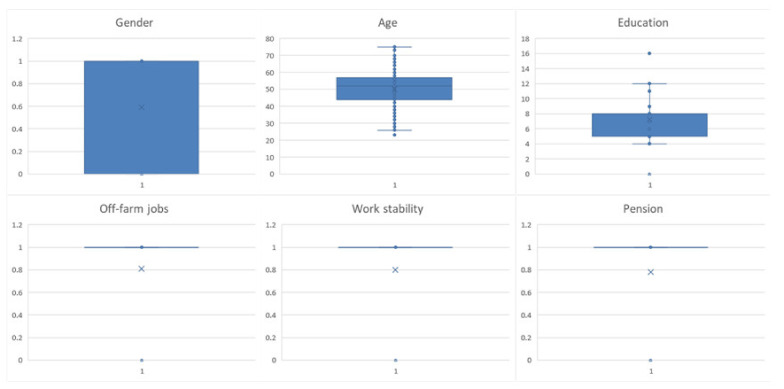
Boxplots of personal characteristics descriptive analysis.

Among the 355 valid questionnaires returned, 293 farmers were willing to transfer land, accounting for 82.54% (Figure 2). According to the dimensions of personal characteristics, the age range of the farmers was 23 to 75 years old, and the years of education ranged from 0 to 16 years. The average age of the farmers who were willing to transfer land was 49.17 years old, and the average for the years of education was 7.71 years. The farmers who were reluctant to transfer land were 5.2 years younger and 2.34 years more educated. It is preliminarily believed that the willingness of older farmers to transfer land is weaker than that of younger farmers, and the higher the education level, the stronger the willingness to transfer land (Figure 3).

**Figure 4:**
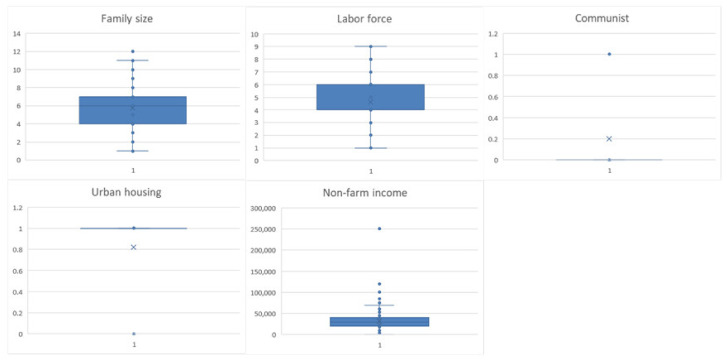
Boxplots of family characteristics descriptive analysis.

According to the dimensions of family characteristics, the average number of households with a willingness to transfer land was 5.83, and the average labor force was 4.61, both of which were more than those without the willingness to transfer land. It is preliminarily believed that the family population and the family labor force are negatively correlated with the farmers’ willingness to transfer land. In the survey, 291 urban farmers bought houses, of which 279 were willing to transfer land, accounting for 95.88%. Therefore, this factor should be positively correlated with farmers’ willingness. The questionnaire data show that the factors of whether there is a party member in the family and the non-agricultural income of the family should also have a positive correlation (Figure 4).

**Figure 5:**
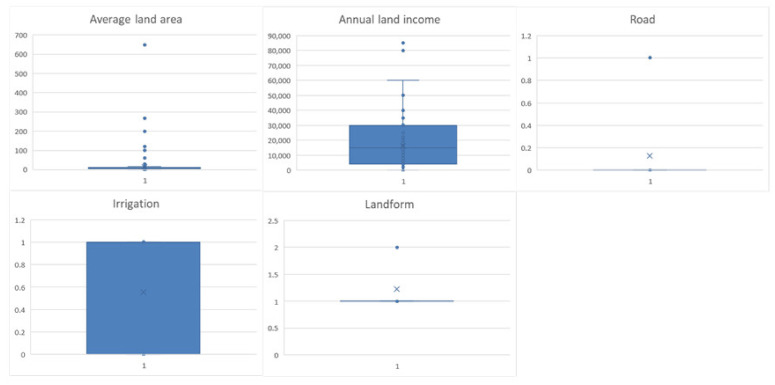
Boxplots of land characteristics descriptive analysis.

According to the land feature dimension, among the 355 farmers who received the questionnaire, 310 believed that their production roads were less convenient, and 196 believed that their irrigation conditions were relatively good. The terrain where 275 respondents cultivated land was in the plain area, and 80 were located in the hills. Among them, the average unit land area of the farmers who were willing to transfer land was 13.94 Chinese Mu, and the average unit land area of farmers who were not willing to transfer land was 7.61 Chinese Mu, which is smaller than the former. In the variable of annual land income, the average annual land income of 293 farmers who were willing to transfer land was CNY 16,941.98, and the average annual income of 62 farmers who were not willing to transfer land was CNY 12,677.42 (Figure 5).

**Figure 6:**
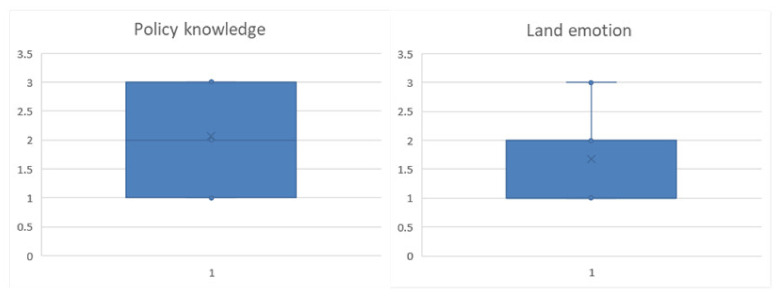
Boxplots of cognitive characteristics descriptive analysis.

According to the cognitive dimension, in terms of the degree of understanding of policy, 95 respondents were very unfamiliar, 144 were familiar, and 116 were very familiar. Among them, 114 were very knowledgeable about policy pertaining to willingness to transfer land, accounting for 38.91%. Two respondents were very familiar with policy, accounting for 3.23%, so it is initially believed that there is a positive correlation between this factor and the willingness of farmers. In terms of the factors associated with emotional connection to the land, 180 farmers had a very weak emotional connection, and 67 farmers had a very heavy emotional connection to the land (Figure 6). Among the farmers who were willing to transfer land, 24 farmers had strong feelings about the land, accounting for 8.19%, and 44 farmers who were not willing to transfer land accounted for 70.97%. Therefore, we preliminarily judge that there is a negative correlation between the variable and farmers’ willingness to transfer land. The descriptive statistics of the model variables are shown in Table 3.

### 3.3. Econometric Model Selection

We chose the sample selection model proposed by Heckman to address sample selection bias [38]. First, we use the Probit model in the first stage to analyze whether the farmers were willing to transfer land and its influencing factors. In this process, we used “$WTT_{i}\;$= 1” to indicate that farmers were willing to transfer land, and “$WTT_{i}\;$= 0” to indicate that farmers were unwilling to transfer land. The observable $WTT_{i}$ is determined by the unobservable latent variable$\;WTT_{i}^{*}$, and the estimated equation is:

(3) $$WTT_{i}^{*}\;=\alpha_{0}\alpha_{i}X_{i}+\sigma \;(i=1,\;2,\;\ldots ,\;n)$$

In the formula, $\alpha_{0}$ represents the constant term, α*_i_* (*i* = 1, 2, …, *n*) represents the regression coefficient of each variable, $X_{i}$ (*i* = 1, 2, …, *n*) represents each explanatory variable, and σ represents the random disturbance term.

Obtain the estimated value α_i_ according to Formula (3), and then calculate the inverse Mills ratio for each sample individual *i*:

(4) $$\bar{\lambda }=\frac{\phi \left(\alpha_{i}X_{i}\right)}{\varphi \left(\alpha_{i}X_{i}\right)}$$

In Formula (4), $\phi \left(\alpha_{i}X_{i}\right)$ and $\varphi \left(\alpha_{i}X_{i}\right)$ represent the density function and cumulative density function of the standard normal distribution of the variables $\alpha_{i}X_{i}$, respectively.

Secondly, the second stage is the result equation, and the OLS model was used for the farmers who were willing to transfer the land to explore the factors that affect the scale of the farmers’ land transferred out.

This stage used the inverse Mills ratio obtained in the first stage as an instrumental variable to correct the sample selection bias in the second stage. The estimated equation obtained after modification is:

(5) $$WTD_{i}=\varepsilon 0+\varepsilon_{i}V_{i}+\varepsilon_{m}\lambda_{m}+\delta \;(i=1,\;2,\;\ldots ,\;n)$$

In Formula (5), $V_{i}$ is the explanatory variable, $WTD_{i}$ is the specific value of the year that the ith farmer is *WTT_i_*, ε*_i_*(*i* = 1, 2, …, *n*) and ε*_m_*are the explanatory variables, and *δ* is the random perturbation term. *λ* is the inverse Mills ratio calculated according to the first stage, which is used to correct the sample selection bias. If *λ* is significantly non-zero, it indicates that there is indeed a selection bias, so the model is valid.

### 3.4. Measurement Results

We tested the multicollinearity for each independent variable. The mean variance inflation factor (VIF) is 2.28. The multicollinearity among the variables is reasonable. Relying on the Heckman selection model and using Stata 15.0 software (StataCorp LLC, College Station, TX, USA), the regression analysis of WTT/WTD was carried out. The χ is 54.82, and the *p*-value is 0.0000. The overall effect is good. The inverse Mills ratio is 4.90, which is significant at 10% of the statistical level. This shows that there is bias in the sample, and the model selection is effective. The measurement results are detailed in Table 4 and Table 5.

Among the factors affecting WTT in the first stage, five factors are significant: farmers’ job stability, whether they buy houses in urban areas, whether the production roads are convenient, their understanding of the government’s land transfer policy, and farmers’ land sentiment. Among them, three factors, including job stability, whether to buy a house in an urban area, and understanding of the government’s land transfer policy, were positively correlated with WTT, and whether the land production road was convenient and farmers’ land sentiment were negatively correlated with WTT. In the second stage, among the factors affecting WTD, six factors were significant: the age of the farmer, the stability of the farmer’s job, the number of people in the farmer’s family, whether to buy a house in an urban area, the non-agricultural income of the family, and land sentiment. Among them, the three factors of farmers’ job stability, whether farmers buy houses in urban areas, and the non-agricultural income of the family were positively correlated with WTD, and the three factors of age, family size, and land sentiment were negatively correlated with WTD.

### 3.5. Measurement Results Analysis

Among the 18 factors affecting farmers’ WTT, five were significant. This shows that the stronger the job stability of the farmers, the worse the transportation convenience, the higher the understanding of land policy, the weaker the land sentiment, and the stronger the farmers’ WTT. At the same time, the WTT of the farmers who bought a house in the city is higher than that of the farmers who have not bought a house in the city. These results are consistent with the original hypothesis. Among the 18 factors affecting farmers’ WTD, six were significant. This shows that the younger the farmer is, the smaller the family population, the higher the non-agricultural income of the family, and the weaker the land sentiment is; the longer the farmer’s WTD is, the more stable the job is, and the WTD of a farmer who buys a house in the city is greater than one who does not.

(1) Interpretation of personal characteristic variables

The impact of the personal characteristic variables on WTT/WTD is consistent with Hypothesis 1.

The older the household head, the lower the WTD. Firstly, when there are more opportunities for young farmers to work or start businesses in cities, the benefits will be far greater than the benefits of agricultural production. Secondly, the young people’s low opinion of farming and rejection of high-intensity agricultural work make WTT extremely strong. Thirdly, affected by age, the job opportunities of migrant workers also decrease as the age increases, especially for farmers after the age of 55, and the WTT is generally lower.

The WTT/WTD of households with stable jobs was stronger than that of households with unstable jobs. Firstly, the job stability of farmers is linked to their expected future income. Farmers with strong job stability usually have a better future expected income and rely less on land pension security. Secondly, farmers with stable jobs have fixed jobs and do not have time to engage in busy farming activities. Thirdly, farmers with stable jobs generally have better work intensity and wage income than agricultural production, so they usually have stronger WTT, and they are more willing to spend their time on non-agricultural work with higher input–output ratios.

(2) Interpretation of family characteristic variables

The impact of family characteristic variables on WTT/WTD is consistent with Hypothesis 2.

The larger the household size, the weaker the WTD. Farmers with a larger number of households usually have a certain proportion of elderly people and children. On the one hand, the existence of a certain proportion of the elderly population and children in the family population means that the family is responsible for supporting the elderly and raising children. Therefore, he cannot leave his family for a long time to seek work, and subsidizing his family through agricultural income and nearby odd jobs has become his last resort. On the other hand, elderly people in their 60s and 70s at home cannot do heavy physical work, but they can help young people with household chores and childcare, which gives young people the energy to cultivate the land.

The WTT/WTD of urban households who bought a house was much higher than that of urban households who did not buy a house. At present, there are generally three situations for farmers who enter the city to buy a house. One is that they have certain economic strength, and they choose to buy a house in the city in order to further improve their family life and quality of life. The second situation is the need for employment and entrepreneurship, and the third is being forced to buy a house because of marriage or children’s schooling needs. Farmers who buy houses in urban areas move away from the land, and agricultural production activities are no longer convenient.

The higher the household’s non-agricultural income, the stronger the farmer’s WTD. The current income of Chinese farmers mainly includes two parts, namely, agricultural income and non-agricultural income. In recent years, with the development of China’s secondary and tertiary industries and township enterprises and the implementation of the national strategy of “mass entrepreneurship and innovation”, farmers have more sources of non-agricultural income than before, and the comparative income from land has gradually declined. The higher the non-agricultural household income, the lower the proportion of agricultural income in the total household income, the less important the land is to farmers, and the higher the WTD.

(3) Interpretation of land characteristic variables

The impact of land characteristic variables on WTT/WTD is consistent with Hypothesis 3.

The worse the road traffic conditions, the stronger the farmers’ WTT. Road traffic conditions determine the accessibility of mechanized equipment and vehicles. Farmers in places inconvenient for mechanized equipment and vehicles to reach can only farm using primitive manual methods, which increases the input of labor in agricultural production, increases production costs, and reduces production. Therefore, the farmers’ WTT in areas with poor road traffic conditions is stronger.

(4) Interpretation of cognitive variables

The impact of cognitive variables on WTT/WTD is consistent with Hypothesis 4.

The deeper the farmer’s understanding of land transfer policy, the stronger the WTT. The more farmers know about the government’s policies on land transfer, the fewer concerns they have about the benefits of land transfer, the procedures for land transfer, and whether the land can be recovered after transfer, and the more confident they are in maintaining land during transfer. The more they understand their own legitimate rights and interests, the more actively they will transfer the land out.

The stronger the farmers’ land sentiment, the weaker the WTT/WTD. China is founded on agriculture, and land is the foundation for farmers to survive. Chinese farmers who have experienced difficult times have a high emotional dependence on land and food. The different feelings of farmers towards the land greatly affect their WTT/WTD. The survey found that some older farmers are still reluctant to transfer land even if they have non-agricultural employment opportunities. Influenced by traditional thinking, they see the land as the last option for security when nothing else is left, and this concept is deeply ingrained in their hearts.

### 3.6. Expected Land Transfer Time

A total of 82.53% of the respondents hoped to transfer land. According to Formula (3) and Table 3, after removing the sample selection bias, the WTD of rural residents is 9.34 years.

## 4. Discussion

It is indeed necessary for China to take measures to increase the willingness of farmers to transfer their land. First of all, compared with the actual situation in China’s rural areas, the current decentralized household contract responsibility system in China has many drawbacks. Secondly, combined with the results of this paper, eight factors, including farmers’ age and job stability, have a significant impact on farmers’ WTT/WTD. Among them, we believe that the five factors of job stability, whether to buy a house in an urban area, family non-agricultural income, degree of policy understanding, and land sentiment are highly guiding and controllable. At the same time, the city of Feicheng investigated in this paper is one of the top 100 economically developed counties, and its population with no stable non-agricultural employment opportunities accounts for 20% [23], and the proportion will further increase in the whole country. Compared with the average level of developed countries, China’s urbanization rate still has a gap of nearly 20% [39]. Combined with the 355 samples of this survey, the average non-agricultural household income is only CNY 32,194.37, which is still far from China’s per capita disposable income of CNY 35,128 in 2021 [2]. Only 32.68% of the farmers had a very good understanding of policy, and only 50.99% of the farmers had a weaker land sentiment. Combined with the data and the above, it is indeed necessary for China to take measures to improve WTT/WTD, and there is great potential for improvement.

It is necessary to fundamentally change the peasants’ overly strong land complex, that is, to change their thinking. The government should take a series of measures to ideologically reverse the excessive dependence of farmers on land sentiment, and combine the new era background so that farmers can correctly understand the land and correctly locate the relationship between farmers and land under the new era background, so as to have the effect of land intensification. Objective functional dependence and subjective emotional attachment together constitute people’s land dependence, which is the land dependence of farmers [40]. Intensity determines people’s willingness and actions to transfer land, and farmers’ emotional attachment to land may become a constraint on land transfer [41]. An emotional connection to the land is a non-material, non-utilitarian, simple value pursuit that is different from the spiritual ideals of literati and literati for farmers with a thick soil complex [42].

Enabling farmers to understand land transfer policy is necessary. Farmers’ doubts and “wait-and-see” attitude towards land transfer can only be resolved if farmers have a sufficient understanding of the national land transfer policy and system. The more farmers know about agriculture-related policies, the stronger their willingness to transfer land [19]. Policy understanding and pension insurance have a high impact on farmers’ willingness to participate in land transfer [43]. Once farmers understand the policy advantages of land transfer, they will increase their WTT [44].

It is necessary for farmers to have stable non-agricultural employment opportunities with relatively high income levels, so that they can still have good income expectations after they leave the land, that is, stable employment. When part-time employment becomes the main source of farmers’ income, they put more effort into part-time jobs instead of farming at home to earn more income, and land is relatively less attractive to them, so they show a relatively strong WTT [45]. Job stability reflects farmers’ future expected income to some extent after surveying six counties and districts in southern Shaanxi. Therefore, farmers with good job stability are more willing to transfer land [46].

Finally, urbanization is the most ideal solution to encourage farmers to leave the land for a long time or even completely. The urbanization of rural areas cannot only improve the conditions of rural economic development and improve the quality of life of rural residents, but also prompt farmers to transfer their land out so that they can be completely separated from it [47]. Urban development and labor transfer provide land supplies for land transfer, and the acceleration of new urbanization is a new opportunity for the development of agricultural modernization [48]. The level of urbanization has the greatest impact on land transfer, and the faster the urbanization develops, the higher the degree of land transfer [49].

Combined with the analysis results, we screened out four guiding and controllable variables as an important focus for promoting the land transfer of Chinese farmers. Based on this, we propose the theory of Chinese farmers’ land transfer, that is “transfer of mind, understanding of policies, stabilization of employment, and urbanization” (MPEU theory). Firstly, with the ideological transformation as the guide, the farmers themselves should change their ideological complex, and fundamentally reverse the farmers’ psychological barriers and resistance to land transfer. Secondly, after changing farmers’ thinking, agriculture-related policies should be popularized, and on the basis of breaking their emotional bond with the land, let the farmers understand the convenience of the relevant policies, thus relieving their concerns and improving the WTT. Through the above two steps, the internal conditions for the farmers’ land to be transferred out are basically satisfied. Thirdly, the stable employment of farmers solves the material foundation after the land is transferred out. So far, the internal and external conditions for the farmers’ land transfer have been met, and the land transfer is feasible and spontaneous. On the basis of realizing the above steps, in order to encourage farmers to relinquish the land completely and permanently, urbanization is an ideal choice.

However, the realization of MPEU theory faces many challenges. First of all, it is difficult to change people’s minds. The Chinese people have suffered from hunger for thousands of years, and the importance of food and land is deeply rooted. This generation of Chinese farmers has also suffered from hunger and has a strong sense of the importance of land. Therefore, there are certain challenges in transforming the entrenched thinking of farmers. Moreover, it is difficult for farmers to understand policies. The grass-roots propaganda cadres are limited by their cognitive level and other aspects, and their work strength needs to be tested. There is also uncertainty about whether farmers will accept the relevant propaganda and whether they have enough trust in village and township grass-roots cadres [34]. How to solve the “last mile” of policy propaganda is the key. Again, stable employment is difficult. China’s economy is still in the process of recovery and development after severe shocks such as the COVID-19 epidemic. Small, medium, and micro enterprises and individual industrial and commercial households are facing difficulties in production and operation, and the task of stabilizing employment is even more arduous [50]. China faces huge difficulties in ensuring national employment. There were 9.09 million graduates from colleges and universities in China in 2021 and 10.76 million in 2022 [51]. For farmers with a low educational level, it will be very difficult to create stable non-agricultural employment. Finally, urbanization needs to be gradually promoted. At present, China’s urbanization rate is 64.72% [52], and the urbanization rate in developed countries is generally above 80%. China’s urbanization still has a lot of room for development, but it faces huge challenges. One is China’s high housing prices that make it too difficult for farmers to move into cities, and another is the difference in lifestyle, which makes older farmers reluctant to change their original way of life.

## 5. Conclusions

Combining the current national conditions of China and the status quo of rural arable land, realizing the transformation of land from fragmentation to intensification is the only way for China to move towards agricultural modernization. We selected Feicheng City, Shandong Province, as the research area, conducted regression analysis on the data by means of questionnaires and key interviews, and identified the influencing factors that can affect and change farmers’ willingness to transfer (WTT) their land and willingness to the duration (WTD) of land transfer. Among them, five factors, including job stability, purchasing houses in urban areas, cultivated land roads, degree of policy understanding, and emotional attachment to the land, can significantly affect the farmers’ WTT. Six factors—age, job stability, number of family members, purchasing houses in urban areas, non-agricultural income, and emotional attachment to the land—can significantly affect the farmers’ WTD. At the same time, we further concluded that the average WTD is 9.34 years. Based on this, we propose the “MPEU theory” of farmers’ land transfer. That is, by allowing farmers to change their minds, understand policies, increase non-agricultural employment rate, and improve the level of urbanization, the farmers’ WTT/WTD can be improved, and the level of land intensification can be improved. Finally, agricultural modernization, peasant citizenization, and rural urbanization will be realized.

Based on the findings of this paper, we make the following policy recommendations.

Firstly, it is necessary to steadily promote land transfer on the basis of respecting the feelings of Chinese farmers towards their land. The government should let farmers fully understand the system design of the “separation of three rights” of land management rights, ownership, and contract rights. The government should establish and improve the land transfer market and related laws and regulations, and protect the legal rights and interests of farmers when the land is transferred out. The farmers who transfer the land can receive rent on time, and the land can be recovered smoothly and on time after the contract expires. This will promote the formation of a land transfer atmosphere in rural areas, forming a psychological follow-up effect.

Secondly, policy guidance should be strengthened to enable farmers to fully understand the land transfer policy. To ensure farmers have a sufficient understanding of agriculture-related policies, the key is to educate village and town cadres. Village and town cadres should go deep into the countryside to reach farmers, carefully answer and summarize the questions and concerns about land transfer that farmers care about, and focus on explaining the issues that farmers are generally concerned about in policy presentations. Combined with the rural situation, the use of leaflets, broadcasts, and other forms, combined with the internet, social media, and other new media to carry out multi-dimensional and multi-channel policy publicity.

Thirdly, the vocational skills of farmers should be cultivated and the level of non-agricultural employment improved. Township governments should, with the support of county-level governments, increase investment promotion efforts, attract more foreign enterprises to invest and set up factories locally, and at the same time provide all-round support for farmers who are willing to start businesses, including loans, taxes, and subsidies. The government should encourage the development of vocational skills training institutions for farmers, provide farmers with a variety of labor skills training, and create conditions for them to obtain stable non-agricultural employment opportunities.

Fourthly, measures should be taken to adapt to local conditions and take the road of new urbanization. For able farmers, encourage them to enter the city. Implement local urbanization for villages with a high population density and large scale, and implement consolidation for villages with a sparse and scattered population. On this basis, build infrastructure and public services, develop industrial support, and then remove farmers from the land and achieve land intensification.

## Figures and Tables

**Table 1:** Questionnaire design and reference.

Question			Options	Objective	Assessment	Reference
Personal characteristics	What is your gender?		male = 1, female = 0	Obtain independent variables for regression analysis	closed	[24,25,26,27]
	What is your age?		number		open-ended	[24,25,26]
	How many years of education have you had?		number		open-ended	[25,28]
	Do you have non-farm payrolls?		yes = 1, no = 0		closed	[26,29]
	Do you have a stable non-farm payroll?		yes = 1, no = 0		closed	[29,30]
	Have you participated in social endowment insurance?		yes = 1, no = 0		closed	[28,30]
Family characteristics	How big is your family?		number	Obtain independent variables for regression analysis	open-ended	[25,31]
	How much labor is there in your household?		number		open-ended	[29,31]
	Are there any Communists in your family?		yes = 1, no = 0		closed	[24]
	Are you buying a house in town?		yes = 1, no = 0		closed	[29,31]
	What is your household’s non-farm income?		number		open-ended	[29,31]
Land characteristics	What is your total household arable land?	Obtain average land area	number	Obtain independent variables for regression analysis	open-ended	[24,31,32]
	What is the number of plots of land in your home?		number		open-ended	[27,31,32]
	What is your annual income from arable land?		number		open-ended	[26,31]
	Is your arable land production road convenient?		yes = 1, no = 0		closed	[24,31]
	Is irrigation of your arable land convenient?		yes = 1, no = 0		closed	[31]
	What is the terrain of your farmland?		plain = 1, hills = 0		closed	[24,26]
Cognitive variables	How well do you understand the land circulation policy?		1 = ignorant—understand = 3	Obtain independent variables for regression analysis	closed	[27]
	How emotionally dependent are you on the land?		1 = light—strong = 3		closed	[33]
	What are your main sources of information about land circulation policies?		1. Government publicity and guidance; 2. Internet, television and other media; 3. Friends; 4. Self-study		closed	[24,26,31]
Willingness to transfer land	Would you rather transfer the land out? (WTT)		yes = 1, no = 0	dependent variable	closed	[25,26,34]
	How many years would you like to transfer your land out? (WTD)		number		open-ended	[26,34]
	If not, why?		statement of reason	cause analysis	open-ended	[26,34]

**Table 2:** Variable definitions and assignments.

Category	Variable Name	Definition and Assignment	Variable Type
Personal characteristics	gender	male = 1, female = 0	virtual variable
	age	according to actual age	continuous variable
	education	according to actual years	continuous variable
	off-farm jobs	yes = 1, no = 0	virtual variable
	work stability	yes = 1, no = 0	virtual variable
	pension	yes = 1, no = 0	virtual variable
Family characteristics	family size	calculate the actual size	continuous variable
	labor force	calculate the actual size	continuous variable
	communist	yes = 1, no = 0	virtual variable
	urban housing	yes = 1, no = 0	virtual variable
	non-farm income	calculate actual income	continuous variable
Land characteristics	average land area	calculate the actual area	continuous variable
	annual land income	calculate actual income	continuous variable
	road	convenient = 1, not = 0	virtual variable
	irrigation	convenient = 1, not = 0	virtual variable
	landform	plain = 1, hills = 0	virtual variable
Cognitive variables	policy knowledge	1 = ignorant—understand = 3	virtual variable
	land emotion	1 = light—strong = 3	virtual variable

**Table 3:** Descriptive analysis.

Category	Variable Name	Mean	Std. Dev.	Median	Min	Max
Dependent variables	WTT	0.83	0.38	1	0	1
	WTD	9.44	6.75	10	0	30
Personal characteristics	gender	0.59	0.49	1	0	1
	age	50.16	11.39	52	23	75
	education	7.30	2.96	8	0	16
	off-farm jobs	0.81	0.39	1	0	1
	work stability	0.80	0.40	1	0	1
	pension	0.78	0.41	1	0	1
Family characteristics	family size	5.78	2.16	6	1	12
	labor force	4.61	1.59	4	1	9
	communist	0.20	0.40	0	0	1
	urban housing	0.82	0.38	1	0	1
	non-farm income	32,194.37	24,345.16	30,000	1000	250,000
Land characteristics	average land area	12.83	41.51	7	0	650
	annual land income	16,197.18	14,296.86	15,000	0	85,000
	road	0.13	0.33	0	0	1
	irrigation	0.56	0.50	1	0	1
	landform	1.23	0.42	1	1	2
Cognitive variables	policy knowledge	2.06	0.77	2	1	3
	land emotion	1.68	0.77	1	1	3

**Table 4:** Probit selection estimates in Heckman sample selection model regression results.

Category	Variable Name	Coef.	Std. Err.
Personal characteristics	gender	0.20	0.48
	age	−0.01	0.02
	education	−0. 05	0.09
	off-farm jobs	0.68	0.74
	work stability	1.85 (**)	0.78
	pension	0.73	0.57
Family characteristics	family size	0.03	0.14
	labor force	−0.08	0.18
	Communist	0.49	0.78
	urban housing	1.41 (**)	0.59
	non-farm income	0.00	0.00
Land characteristics	average land area	0.02	0.05
	land income	0.00	0.00
	road	−1.12 (*)	0.64
	irrigation	0.03	0.54
	landform	−0.90	0.73
Cognitive variables	policy knowledge	0.76 (*)	0.41
	land emotion	−0.98 (**)	0.39

Note: *p*-values in parentheses **, * represent 5%, and 10% significance levels, respectively.

**Table 5:** Outcome estimates in Heckman sample selection model regression results.

Category	Variable Name	Coef.	Std. Err.
Personal characteristics	gender	0.18	0.68
	age	−0.07 (**)	0.03
	education	−0.03	0.12
	off-farm jobs	−0.95	2.05
	work stability	4.35 (**)	1.99
	pension	1.65	1.25
Family characteristics	family size	−0.55 (***)	0.19
	labor force	0.17	0.27
	Communist	0.89	1.19
	urban housing	4.15 (***)	1.51
	non-farm income	0.00 (**)	0.00
Land characteristics	average land area	0.00	0.01
	land income	0.00	0.00
	road	1.25	1.31
	irrigation	0.02	0.72
	landform	−0.23	1.19
Cognitive variables	policy knowledge	0.20	0.52
	land emotion	−1.39 (**)	0.58

Note: *p*-values in parentheses, ***, ** represent 1%, 5% significance levels, respectively.

## Data Availability

The data presented in this study are available on request from the corresponding author.
